# Supplementary material for: Type I Diabetes in Zebrafish Reduces Sperm Quality and Increases Insulin and Glucose Transporter Transcripts
Source: Int J Mol Sci. 2023 Apr 11;24(8):7035. doi: 10.3390/ijms24087035 (PMC10138585; doi:10.3390/ijms24087035)
Supplement: Supplementary file 1 [file ijms-24-07035-s001.zip › ijms-2306219-supplementary.pdf]

Supplementary Table S1. Forward and reverse primers used for target genes analysis through quantitative real-time polymerase chain reaction (qPCR).

Target Gene	Gene Name	Forward Primer (5′-3′)	Reverse Primer (5′-3′)	Annealing Temperature (°C)
ef1a	elongation factor 1 alpha	AGCCCCTCCTGGCTTTCACCC	TGGGACGAAGGCAACACTGGC	60
insa	preproinsulin	CATTCCTCGCCTCTGCTTC	TGCCTGGGTTAGTGCTTACA	60
insra	insulin receptor a	TCTACAGCGAGGAAAACAAGC	AGAGATAAGATGCGTCCGTTTT	60
slc2a2	solute carrier family 2 member 2	GCCATAACAGCAGGACTACT	GATGACAGACCACAGTACAATCC	60
b4galt2	1,4- galactosyltransferase	ACCATCTTCTGCCTAGCACCA	GAGTATATTTCCCGCCTTCTGTGAC	60
hif1a	hypoxia-inducible factor-1a	CAGCCTTTTTGTGAAGGGGC	TGACTGGAGAAACAGTCCGC	60

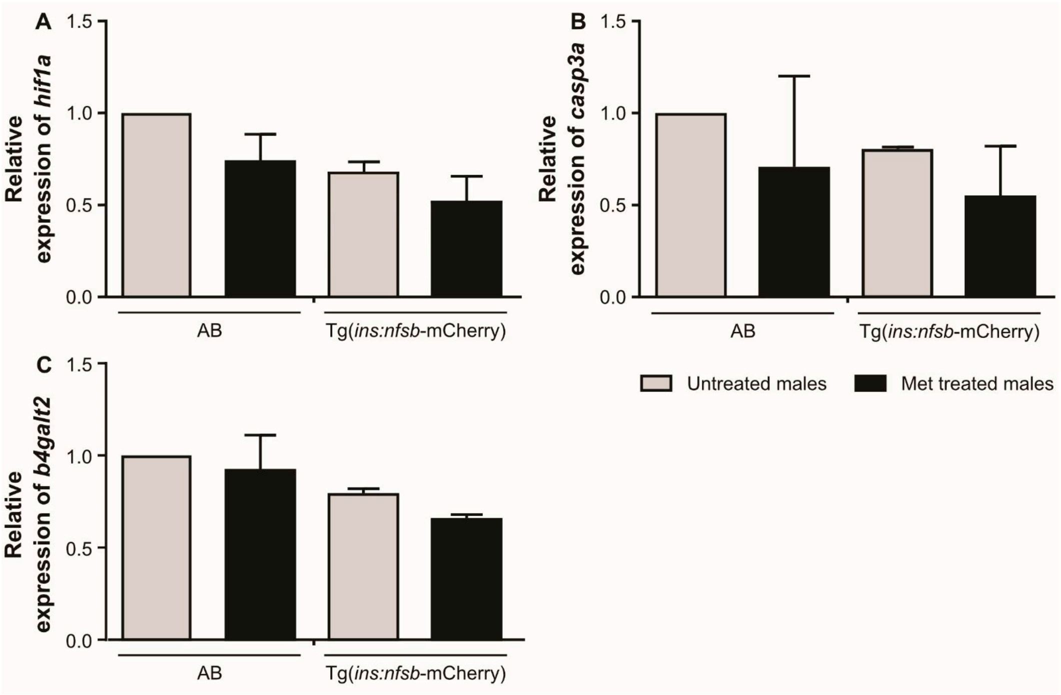

Supplementary Figure S1. Genes relative expression to *ef1α* in zebrafish sperm pools using 2−ΔΔCt method: (**A**) *hif1a* in spermatozoa pools of AB males in control (n = 2) and Met treatment (n = 2), and Tg(*ins*:*nfsb-mCherry*) in control (n = 2) and Met treatment (n = 2) and (**B**) *caspase 3a (casp3a)* in spermatozoa pools of AB males in control (n = 2) and Met treatment (n = 2), and Tg(*ins*:*nfsb-mCherry*) in control (n = 2) and Met treatment (n = 2) and (**C**) *b4galt2* in spermatozoa pools of AB males in control (n = 2) and Met treatment (n = 2), and Tg(*ins*:*nfsb-mCherry*) in control (n = 2) and Met treatment (n = 2) The values plotted represent means ± SD.

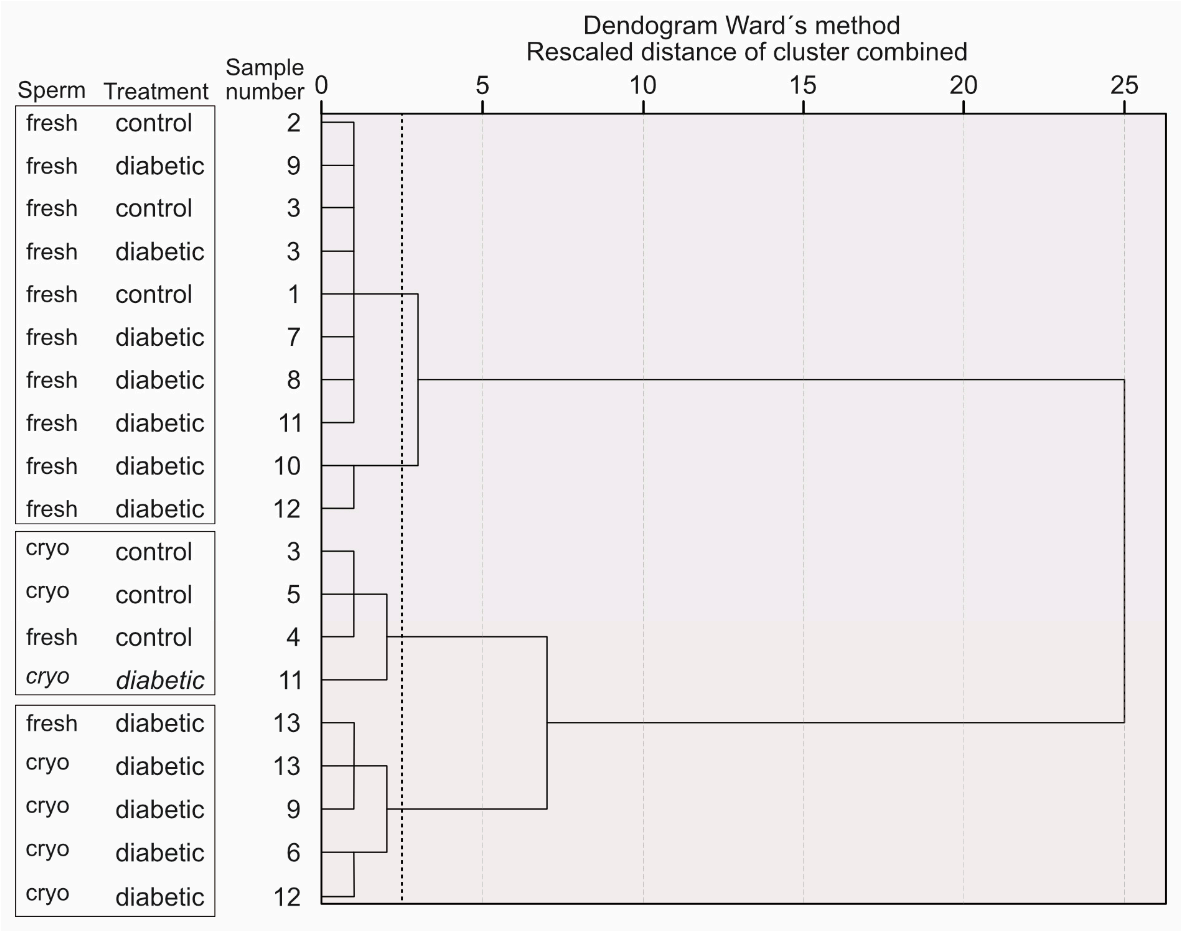

Supplementary Figure S2. Dendogram of Ward´s hierarchical cluster analysis for fresh and cryopreserved sperm (of principal components resulting from motility and DNA fragmentation data) of zebrafish sperm cryopreserved with a −10 °C/min cooling rate. Rectangles discriminate clusters of samples within fresh and cryopreserved sperm from untreated and Met treated Tg(*ins:nfsb-mCherry*) males.
